# Sex-differential cognitive performance on MCCB of youth with BD-II depression

**DOI:** 10.1186/s12888-024-05701-7

**Published:** 2024-05-07

**Authors:** Dong Huang, Shunkai Lai, Shuming Zhong, Yiliang Zhang, Jiali He, Shuya Yan, Xiaosi Huang, Xiaodan Lu, Manying Duan, Kailin Song, Kaiwei Ye, Yandi Chen, Suiyi Ye, Jiankang Lai, Qilin Zhong, Xiaodong Song, Yanbin Jia

**Affiliations:** 1 Department of Psychiatry, First Affiliated Hospital of Jinan University, Guangzhou, 510630 China; 2 School of Management, Jinan University, Guangzhou, 510316 China

**Keywords:** BD-II depression, Sex differences, MCCB, Cognitive deficits, Youth

## Abstract

**Background:**

Recent evidences have shown sex-differential cognitive deficits in bipolar disorder (BD) and differences in cognitions across BD subtypes. However, the sex-specific effect on cognitive impairment in BD subtype II (BD-II) remains obscure. The aim of the current study was to examine whether cognitive deficits differ by gender in youth with BD-II depression.

**Method:**

This cross-sectional study recruited 125 unmedicated youths with BD-II depression and 140 age-, sex-, and education-matched healthy controls (HCs). The Chinese version of the Measurement and Treatment Research to Improve Cognition in Schizophrenia (MATRICS) Consensus Cognitive Battery (MCCB) was used to assess cognitive functions. Mood state was assessed using the 24-item Hamilton Depression Rating Scale (24-HDRS) and the Young Mania Rating Scale (YMRS). Multivariate analysis of covariance (MANCOVA) was conducted.

**Result:**

​Compared with HCs, patients with BD-II depression had lower scores on MCCB composite and its seven cognitive domains (all *p* < 0.001). After controlling for age and education, MANCOVA revealed significant gender-by-group interaction on attention/vigilance (F = 6.224, df = 1, *p* = 0.013), verbal learning (F = 9.847, df = 1, *p* = 0.002), visual learning (F = 4.242, df = 1, *p* = 0.040), and composite (F = 8.819, df = 1, *p* = 0.003). Post hoc analyses suggested that males performed worse in the above-mentioned MCCB tests than females in BD-II depression.

**Conclusion:**

Our study demonstrated generalized cognitive deficits in unmedicated youths with BD-II depression. Male patients performed more serious cognitive impairment on attention/vigilance, verbal learning, and visual learning compared to female patients.

**Supplementary Information:**

The online version contains supplementary material available at 10.1186/s12888-024-05701-7.

## Introduction

Bipolar disorder (BD), characterized by recurring episodes of depression and mania, is considered to be a serious and chronic mental illness. ​BD accounts for 4.1% of the approximately 1 billion people worldwide who suffer from mental illness [1]. According to WHO, BD is the leading cause of global burden of illness and cause 6.5% of all mental disorders-related disability-adjusted life years (DALYs) in 2019 [1]. Among all mental disorders, BD has the highest rate of completed suicides [2, 3]. The suicide rate in patients with BD is approximately 20–30 times greater than in the general population, with the highest risk occurring during depressive episodes [4, 5].

Enduring cognitive deficit has been reported in patients with BD [6–8]. These cognitive deficits include, but are not limited to, sustained attention, executive function, verbal or non-verbal memory and social cognition [9–13]. Moreover, cognitive impairments in BD do not appear to be explained by symptom fluctuations and have been shown to persist after remission of affective symptoms [6, 14–16]. Therefore, it has been considered inherent to BD and independent of the affective state [14]. More than that, cognitive impairment may lead to poor medication adherence and increase the risk of recurrence in BD [17]. Furthermore, cognitive deficits contribute to poor occupational and social functioning, which can lead to poor quality of life and psychosocial disabilities in BD patients [18, 19]. Thus, identifying and intervening early on cognitive deficits caused by BD are crucial to the comprehensive rehabilitation of the illness.

Experimental studies have shown gender-specific effects on cognitive deficits in BD, but the results are mixed. For example, Carrus and colleagues found that male patients had a worse immediate memory than female patients in BD [20]. One study showed that males performed better than females on spatial working memory in both BD patients and healthy subjects [21]; however, another study reported that this sex-specific effect existed in healthy subjects but was not sustained in BD patients [22]. A further shortcoming of previous studies is the use of a small battery of neurocognitive tests that cannot access a full range of cognitive functions. Recently, one study employed a relatively comprehensive neuropsychological battery (i.e., the Repeatable Battery for the Assessment of Neuropsychological Status (RBANS) and the Stroop color-word test) to evaluate gender differences in cognition in BD [23]. The results revealed that males with BD had greater impairment in attention and delayed memory than females, whereas there was no sex effect on other cognitive domains, including immediate memory. Nonetheless, an important limitation of this study was the lack of analysis stratified by BD subtypes, because it is possible that the sex-specific effect on neurocognitive dysfunctions may differ by BD subtypes. Unfortunately, ​in terms of previous studies on gender-differential cognitive performance, most were for BD patients while partly were only for BD subtype I (BD-I) patients; there has been little study devoted to BD subtype II (BD-II) patients.

Studies have confirmed the group differences in cognitive function between BD-I and BD-II subjects. The earlier research found that BD-I patients performed worse global cognition, processing speed, and visual/verbal memory than BD-II patients although the differences were small [24]. The recent studies also showed that BD-I patients might have worse and more widespread cognitive impairments compared to BD-II patients [25, 26], but the latter could experience more severe executive function impairment [27]. Overall, cognitive deficits differed across BD subtypes. Hence, there is a need for further studies to classify distinct types of BD, which may help in providing a scientific basis for precise therapy of cognitive deficits in BD.

Given the lack of research on sex-differential cognitive deficits in young adult patients with BD-II and the more severe deficits in neurocognition during the depressive episode of BD [28, 29], the current study would recruit unmedicated young adult patients with BD-II depression. The aim of this study was to investigate whether cognitive functions were impaired and whether the cognitive impairments were affected by gender in unmedicated young adults with BD-II depression. We hypothesized that the neurocognition declined and the cognitive decline differed by sex in unmedicated young adults with BD-II depression.

## Methods

### Subjects

A total of 125 youths/young adults, aged between 17 and 30 [30], who met Diagnostic and Statistical Manual of Mental Disorders, Fifth Edition (DSM-5) criteria for bipolar disorder type II depression were recruited from the psychiatry department of First Affiliated Hospital of Jinan University in Guangzhou, China. The diagnosis was confirmed by two experienced psychiatrists using the Structured Clinical Interview for DSM-IV (SCID). All patients were in a current depressive episode, with a score of greater than 20 on the 24-item Hamilton Depression Rating Scale (24-HDRS) and less than 7 on the Young Mania Rating Scale (YMRS). In addition, laboratory tests, complete medical history and physical examinations were conducted on the participants. All patients were drug naive or unmedicated for at least 6 months at the time of enrollment. The major criteria for exclusion were: (1) comorbid with other serious psychiatric disorders, such as autism and mental retardation; (2) comorbid with severe somatic illness or organic brain diseases (diabetes, traumatic brain injury, etc.); (3) having history of nonpharmacological therapy (e.g., psychotherapy, repetitive transcranial magnetic stimulation intervention or electroconvulsive therapy) within the 6 months before enrollment; (4) having history of alcohol/substance abuse or dependence; and (5) being pregnant or lactating.

We also recruited 140 age-, sex- and education-matched healthy controls (HCs) from the local community by advertisements. HCs were carefully screened for mental illness with the Structured Clinical Interview for DSM-IV Nonpatient Edition (SCID-NP). The exclusion criteria were the same as for patients. All participants were right-handed and Chinese Han ethnicity.

This study received ethics approval from the Ethics Committee of First Affiliated Hospital of Jinan University, China. Informed consent was obtained from all participants after a detailed introduction and explanation of the study procedures.

### Symptomatic assessment

We collected demographic characteristics and medical history of the participants using standardized questionnaires. The severity of depressive and manic symptoms was assessed with the 24-HDRS and YMRS [31] by two experienced psychiatrists who has received the consistency training (Kappa > 0.8).

### Cognitive measure

Cognitive function was assessed using the Chinese version of the Measurement and Treatment Research to Improve Cognition in Schizophrenia (MATRICS) Consensus Cognitive Battery (MCCB). The applicability of MCCB in BD has been confirmed [32]; MCCB is recommended for evaluating cognitive function in BD [33]. The clinical validity and test-retest reliability of the Chinese version of MCCB in healthy controls and BD patients were established [34, 35]. The MCCB comprises 9 tests, which measure seven cognitive domains: speed of processing (assessed by Brief Assessment of Cognition in Schizophrenia: Symbol coding, Trail Making Test Part A and Category fluency: Animal Naming), attention ⁄ vigilance (assessed by Continuous Performance Test-Identical Pairs), working memory (assessed by Wechsler Memory Scale Spatial span), verbal learning (assessed by Hopkins Verbal Learning Test), visual learning (assessed by Brief Visuospatial Memory Test), reasoning and problem solving (assessed by Neuropsychological Assessment Battery: Mazes), and social cognition (assessed by Mayer-Salovey-Caruso Emotional Intelligence Test: Managing Emotions). The MCCB was performed by eight graduate students who had received special training for consistency, taking approximately 70 min to complete. The inter-rater correlation coefficient of MCCB was over 0.8 after training.

### Statistical analysis

IBM SPSS version 24.0 (SPSS Inc., Chicago, IL, USA) was conducted to all statistical analyses. Continuous variables were expressed as mean ± standard deviation and categorical variables as numbers. Data normality was tested with the Kolmogorov-Smirnov (K-S) test. A two-tailed *p* < 0.05 was considered statistically significant.

Variables of demographics and clinical data were non-normally distributed. Consequently, Mann Whitney U-test was used for group differences analyses of continuous variables and Chi-square tests for categorical variable analyses.

Cognitive variables were almost normally distributed. Group comparison was performed using Student’s t-test and analysis of covariance (ANCOVA) with age and education as covariates. To account for sex differences, multivariate analyses of covariance (MANCOVA) were applied controlling for age and education. Dependent variables were the MCCB composite score and its seven cognitive domains scores; independent variables were sex (male vs. female), group (BD-II depression vs. HCs) and sex by group interaction. Post hoc analyses were performed using a two-way analysis of variance (ANOVA). Pearson’s correlation was used to examine the association of the MCCB with clinical features in patients with BD-II depression, and the association among MCCB and its subscales separately in patients with BD-II depression and HCs, with Bonferroni correction to adjust for multiple testing.

## Results

### Demographic and clinical data

A total of 125 unmedicated patients with BD-II depression (72 females, 57.6%) and 140 HCs (78 females, 55.7%) were recruited. Table 1 shows the demographic data, 24-item HDRS sore and YMRS score. The mean age of the patients with BD-II depression and the HCs were 22.08 ± 3.40 and 22.49 ± 3.36 years old, respectively. There were no significant differences in age, sex and years of education between these two groups (all *p* > 0.05). Moreover, patients with BD-II depression presented with significantly higher 24-item HDRS score than HCs (*p* < 0.001). Besides, no significant sex differential effect on age, years of education or symptomatic score was observed in either the BD-II depression group or the HCs group (Table 2).

**Table 1:** Demographic and clinical data of participants [mean (SD)]

Demographic	BD	HCs	*χ*^2^/z	*p*
Number of subjects	*N* = 125	*N* = 140	–	–
Sex (male/female)	53/72	62/78	0.096	0.757
Age (year)	22.08 ± 3.40	22.49 ± 3.36	-1.083	0.279
Education (year)	14.58 ± 1.93	14.94 ± 1.93	-1.799	0.072
Number of episodes	3.10 ± 1.26	–	–	–
Duration of illness (month)	38.82 ± 34.33	–	–	–
24-HDRS	25.98 ± 4.84	1.52 ± 2.20	-14.247	<0.001
YMRS	2.55 ± 1.71	0.21 ± 0.75	-12.044	<0.001

*BD* Bipolar disorder, *HCs* Healthy controls, *24-HDRS* 24-item Hamilton Depression Rating Scale, *YMRS* Young Mania Rating Scale

**Table 2:** Comparison of sex differences on demographic and clinical data of participants [mean (SD)]

Demographic	BD		*P*-value	HCs		*P*-value
	Male (*n* = 53)	Female (*n* = 72)		Male (*n* = 62)	Female (*n* = 78)	
Age (year)	22.02 ± 3.42	22.13 ± 3.42	0.835	23.06 ± 3.50	22.03 ± 3.20	0.074
Education (year)	14.51 ± 1.76	14.64 ± 2.05	0.775	14.97 ± 2.19	14.92 ± 1.71	0.809
Number of episodes	3.32 ± 1.38	2.94 ± 1.14	0.104	–	–	–
Duration of illness (month)	37.87 ± 30.38	39.53 ± 37.17	0.687	–	–	–
24-HDRS	26.75 ± 5.52	25.42 ± 4.23	0.268	1.21 ± 1.86	1.77 ± 2.43	0.213
Y-MRS	2.47 ± 1.60	2.61 ± 1.80	0.639	0.13 ± 0.53	0.21 ± 0.71	0.407

*BD* Bipolar disorder, *HCs* Healthy controls, *24-HDRS* 24-item Hamilton Depression Rating Scale, *YMRS* Young Mania Rating Scale

### Group differences in cognitive performance

Table 3 presents the comparison of the MCCB composite and its seven cognitive domains scores between unmedicated patients with BD-II depression and HCs. ​In neuropsychological testing with MCCB, patients with BD-II depression performed worse than HCs in all cognitive domains (speed of processing: F = 0.750, df = 263, *p* < 0.001; attention/vigilance: F = 5.901, df = 263, *p* < 0.001; working memory: F = 0.042, df = 263, *p* < 0.001; verbal learning: F = 1.870, df = 263, *p* < 0.001; visual learning: F = 14.663, df = 263, *p* < 0.001; reasoning problem solving: F = 0.299, df = 263, *p* < 0.001; social cognition: F = 10.220, df = 263, *p* < 0.001; composite: F = 2.485, df = 263, *p* < 0.001). After controlling for age and education using ANCOVA, the comparison results of the above-mentioned cognitive functions between patients with BD-II depression and HCs remained consistent and statistically robust (all *p* < 0.001).

**Table 3:** MCCB performance by group using T- and Z-scores [mean (SD)]

Cognitive domains	Mean T-scores (SD)		*F*	*df*	*P*	Adjusted *P*-value
	BD	HCs				
SOP	46.16 ± 7.80	55.06 ± 7.34	0.750	263	<0.001	<0.001
ATT	47.05 ± 8.28	52.74 ± 6.14	5.901	263	<0.001	<0.001
WM	46.63 ± 10.03	53.02 ± 10.07	0.042	263	<0.001	<0.001
VER	46.30 ± 7.82	52.39 ± 6.60	1.870	263	<0.001	<0.001
VIS	44.82 ± 9.73	52.97 ± 6.85	14.663	263	<0.001	<0.001
RPS	47.90 ± 8.70	53.97 ± 8.78	0.299	263	<0.001	<0.001
SOC	44.10 ± 11.39	54.05 ± 8.92	10.220	263	<0.001	<0.001
COS	43.95 ± 7.41	55.19 ± 6.34	2.485	263	<0.001	<0.001

*BD* Bipolar disorder, *HCs* Healthy controls, *SD* Standard deviation, SOP Speed of processing, *ATT* Attention/vigilance, *WM* Working memory, *VER* Verbal learning, *VIS* Visual learning, *RPS* Reasoning problem solving, *SOC* Social cognition, *COS* Composite

### Sex differences in cognitive performance

When the MCCB test and its seven subtests were analyzed by MANCOVA, controlled for age and education, a significant main effect of group (BD-II depression vs. HCs) was still found (Table 4). Post hoc analyses showed lower scores of the MCCB test and its seven subtests in male patients with BD-II depression than in male HCs (all *p* < 0.001), with similar results among female subjects (speed of processing: *p* < 0.001; attention/vigilance: *p* = 0.001; working memory: *p* = 0.003; verbal learning: *p* = 0.001; visual learning: *p* < 0.001; reasoning problem solving: *p* < 0.001; social cognition: *p* < 0.001; composite: *p* < 0.001). Furthermore, MANCOVA revealed statistically significant interaction between gender and group for MCCB attention/vigilance (F = 6.224, df = 1, *p* = 0.013), verbal learning (F = 9.847, df = 1, *p* = 0.002), visual learning (F = 4.242, df = 1, *p* = 0.040) and composite (F = 8.819, df = 1, *p* = 0.003) domains. Post hoc analyses suggested that female patients had higher scores in attention/vigilance (*p* < 0.001), verbal learning (*p* = 0.001), visual learning (*p* = 0.006), and composite (*p* = 0.001) than male patients. However, MANCOVA indicated no significant group-by-sex interaction or main effects of sex for speed of processing, working memory, reasoning problem solving, and social cognition.

**Table 4:** Comparison of sex differential on cognitive function of T-scores

Cognitive domains	BD		HCs		Group	Gender	Group*gender
	Male	Female	Male	Female	*P*-value	*P*-value	*P*-value
SOP	45.60 ± 7.93	46.57 ± 7.74	55.53 ± 7.45^+^	54.69 ± 7.27^+^	<0.001	0.947	0.338
ATT	43.91 ± 9.55^*^	49.36 ± 6.33	52.11 ± 6.87^+^	53.23 ± 5.48^+^	<0.001	<0.001	0.013
WM	45.19 ± 9.88	47.69 ± 10.07	53.52 ± 10.36^+^	52.63 ± 9.88^+^	<0.001	0.517	0.175
VER	43.91 ± 8.67^*^	48.06 ± 6.76	53.15 ± 7.10^+^	51.78 ± 6.16^+^	<0.001	0.114	0.002
VIS	42.43 ± 10.18^*^	46.57 ± 9.06	53.02 ± 7.27^+^	52.94 ± 6.54^+^	<0.001	0.049	0.040
RPS	48.09 ± 8.84	47.76 ± 8.66	54.90 ± 7.31^+^	53.23 ± 9.78^+^	<0.001	0.358	0.538
SOC	43.45 ± 10.81	44.58 ± 11.85	54.40 ± 9.57^+^	53.77 ± 8.43^+^	<0.001	0.845	0.486
COS	41.66 ± 7.87^*^	45.64 ± 6.61	55.74 ± 6.76^+^	54.74 ± 5.99^+^	<0.001	0.077	0.003

*BD* Bipolar disorder, *HCs* Healthy controls, *SD* Standard deviation, *SOP* Speed of processing, *ATT* Attention/vigilance, *WM* Working memory, *VER* Verbal learning, *VIS* Visual learning, *RPS* Reasoning problem solving, *SOC* Social cognition, *COS* Composite

^*^Within-group comparison of males and females in patients or in healthy controls: ^*^p < 0.05

^+^Between-group comparison of patients and healthy controls in males or females: ^+^*p* < 0.05

We further investigated the relationship between attention/vigilance, verbal learning, visual learning and composite in patients and HCs, respectively. In patients with BD-II depression, composite was positively associated with attention/vigilance (*r* = 0.517, *P*_Bonferroni_ < 0.001), verbal learning (*r* = 0.471, *P*_Bonferroni_ < 0.001), and visual learning (*r* = 0.423, *P*_Bonferroni_ < 0.001), while verbal learning was also positively linked to visual learning (*r* = 0.347, *P*_Bonferroni_ < 0.001), with no relationship between attention/vigilance and either verbal learning or visual learning (Supplementary Material, Figure S1). Similar results were obtained after Bonferroni correction in HCs (Supplementary Material, Figure S2).

### Relationship between cognition and clinical features in female and male patients

As shown in Table 5, the associations between cognitive functions and clinical index were analyzed separately in female and male patients with BD-II depression. In female patients, Pearson’s correlation analyses found significant negative relationships between MCCB speed of processing score and duration of illness (*r* = − 0.235, *p* = 0.047), MCCB verbal learning score and number of episodes (*r* = − 0.289, *p* = 0.014), as well as MCCB composite score and 24-HDRS score (*r* = − 0.250, *p* = 0.034). However, only the association between MCCB verbal learning score and number of episodes passed the Bonferroni correction (*p* < 0.05/3 = 0.017). Conversely, no significant correlations were determined in male patients between any of the cognitive domains and clinical characteristics.

**Table 5:** Correlations between clinical data and MCCB in BD patients

Cognitive domains	Male			Female		
	NE	DI	24-HDRS	NE	DI	24-HDRS
SOP	0.180	0.021	0.114	-0.180	**-0.235** ^*^	-0.091
ATT	-0.078	-0.184	-0.014	-0.064	-0.008	-0.049
WM	-0.126	-0.179	0.167	0.082	0.106	0.075
VER	-0.074	-0.082	0.177	**-0.289** ^*^	-0.143	0.024
VIS	0.008	0.098	-0.072	-0.097	-0.038	-0.027
RPS	-0.109	0.056	0.032	-0.163	-0.020	-0.083
SOC	-0.025	0.090	0.117	-0.122	-0.173	0.045
COS	-0.057	-0.052	0.138	-0.080	-0.075	**-0.250** ^*^

*NE* Number of episodes, *DI* Duration of illness, *24-HDRS* 24-item Hamilton Depression Rating Scale, *SOP* Speed of processing, *ATT* Attention/vigilance, *WM* Working memory, *VER* Verbal learning, *VIS* Visual learning, *RPS* Reasoning problem solving, *SOC* Social cognition, *COS* Composite

The bold contents have statistically significant

^*^p < 0.05

## Discussion

The current study is one of the few studies to explore gender differences in cognitive dysfunction among patients with BD. ​The present study provides a scientific rationale for sex-differential cognitive impairment in BD-II depression. ​As far as we know, this is the first case-control study to investigate the gender-specific effects of neurocognition in youth/young adult patients with BD-II depression. Our study has three major findings: firstly, patients with BD-II depression had lower scores on all MCCB cognitive domains compared to HCs; secondly, males patients obtained fewer points than females in MCCB tests of attention/vigilance, verbal learning, visual learning, and composite; thirdly, the MCCB verbal learning score was negatively associated with the number of episodes in female patients with BD-II depression.

In the current study, we found that youth patients with BD-II depression performed worse than HCs in all MCCB cognitive domains. These findings were in line with previous meta-analyses of cognitive performance on MCCB in adult patients with BD [32]. Furthermore, one study using the Repeatable Battery for the Assessment of Neuropsychological Status (RBANS) to measure cognitions also indicated multidomain cognitive impairments of adult patients with BD-II depression [36]. Together with previous findings, the current study results suggested a wide range of cognitive deficits in patients with BD-II depression. The widespread cognitive decline may be involved in multiple pathological mechanisms in patients with BD-II depression. Prior research has found that anomalous neuronal function and metabolism, aberrant neuroendocrine system, inflammation, etc., play an important role in the pathogenesis of cognitive deficits in depressed patients with BD-II. For example, our previous studies have revealed an association between abnormal neuronal connectivity/neurometabolism and cognitive dysfunction in patients with BD-II depression [37, 38]. There is also evidence that serum brain-derived neurotrophic factor (BDNF), including its levels and gene polymorphism, contribute to the mechanism of cognitive deficits in BD-II depression [36, 39]. Additionally, abnormalities in the neuroendocrine system (e.g., corticosteroids and thyroid hormone) are possible causes of impairments in multiple cognitive domains in BD-II depression [40, 41]. Moreover, ​appreciation of the role of inflammation in the cognitive deficits of BD-II depression is widely recognized and better understood [42–45].

The results of the present study illustrated gender differences in neurocognitive performance in youth patients with BD-II depression. Specifically, male patients performed worse cognition compared to male healthy controls, as did female patients related to female healthy controls; male patients also had more severe cognitive impairment than female patients in the domains of attention/vigilance, verbal learning, and visual learning. In healthy controls, however, no significant sex-divergent effects on cognitive performance were observed. Additionally, verbal learning showed a positive correlation with visual learning, but none of them were related to attention/vigilance. In other word, loss of attention and learning ability was more profound in male patients than female patients. These results agreed with prior studies on cognitive function [23, 46, 47], which implicated that gender is a potential moderator for cognitive deficits in patients with BD-II depression. However, the subjects of most previous research on sex-differential cognition in BD have been in a wide age span, from young to middle-aged adults. As we all known, cognitive function tends to decline with advancing age. Thus, the large age span might have an impact on the reliability of the results of the previous studies on gender differences in cognition in BD. The current study, which recruited only young adults aged 17–30, can make up for this shortcoming. Nonetheless, the consistent findings from our study and previous studies further support the gender effect on cognitive deficits in BD-II depression.

The underlying mechanism by which gender affects neurocognition in patients with BD-II depression is complex and likely multifactorial. Cerebral sex dimorphism has been suggested to be associated with sex-related cognitive deficits. Jogia et al. [48] found that women with BD had increased activation in the left caudate during decision-making task and reduced activation in the right ventrolateral prefrontal cortex during fearful facial affect categorization task compared to men with BD, while these affected brain regions are related to emotional and cognitive regulation. Notably, the anterior cingulate cortex is a key region for cognitive function in BD [49–51]. Many studies have reported significant volume decline in the left anterior cingulate cortex in patients with BD [52–54]; however, recent research stratifying the analysis by sex revealed that the volume reduction in left anterior cingulate cortex is only observed in male BD patients [55]. Furthermore, the increased thickness of the right anterior cingulate cortex occurred only in male patients with first-episode BD [56]. Mitchell et al. [57] also showed that in adolescent patients with BD, males had a smaller left supramarginal gyrus and right inferior parietal lobule than females, which were link to cognitive impairments. Taken together, sexually dimorphic in brain structural and functional abnormalities may be a plausible mechanism underlying sex-based effect on neurocognition in BD-II depression. Usually, the sex dimorphism of brain structures is correlated with the effects of sex hormones [58]. Sex hormones, particularly estrogen, may play an important role in the sexual dimorphism in cognitive impairment of BD-II depression [59]. Estrogens can either directly act on multiple brain regions, including the hippocampus and prefrontal cortex, to participate in cognitive function or improve neurocognition by modulating multiple brain neurotransmitter systems, including dopamine, serotonin and glutamate [60–62]. Research has further shown that estrogens have neuroprotective and anti-inflammatory effects and are important for women’s health and neurocognition [63–66]. In female patients with BD, estrogens can regulate BDNF concentrations of multiple brain regions such as the hippocampus, mitigate oxidative stress, and deactivate inflammatory response, which may be the underlying mechanism of better neurocognitive performance in female patients with BD [59]. Apart from that, the expression of gender disparity in cognitive function may be influenced by genetic factors [67]. A prior study investigating the sex-specific role of three BDNF single-nucleotide polymorphisms (SNPs) (rs6265, rs7103411, and rs7124442) in cognitive aging showed that there was only a relationship between rs6265 and processing speed in females and no correlation between BDNF SNPs and cognition in males [68]. ​A recent animal study also reported that the multigenerational inheritance of the relationship between histone modifications and age-associated cognitive decline was sex-specific [69]. In addition, others have concentrated on the potential impact of inflammation. It appears that sex-related variability in exacerbated inflammation may underlie sex-mediated differential cognitive decline in BD-II depression. ​For instance, one study found that immediate verbal learning and delayed verbal learning performed better and were negatively related to C-reactive protein (CRP) concentrations in female patients with BD, whereas no correlation was observed between cognition and CRP in male patients with BD [46]. Another study demonstrated upregulated Suppressors of cytokine signaling (SOCS) genes only in male patients with BD [70], while inflammation has certain causal relationships with cognitive function [71]. Overall, female patients performed better in multiple cognitive domains than male patients in BD-II depression, which may be linked to brain structure and function, sex hormones, genetic factors, and inflammation. Unfortunately, few studies have examined the gender-differential cognitive deficits in BD, the gender-differential cognitive impairment across subtypes of BD, and the gender-differential cognitive dysfunction in BD-II. ​Future and more in-depth studies are needed to address the sex-specific effect on neurocognition and the underlying mechanisms in BD-II depression.

The current study also revealed an inverse correlation between MCCB verbal learning score and number of episodes in female patients, which implied that illness recurrence may impair the verbal learning in BD-II depression female patients. Previous study has reported multi-episode patients with BD performed poorly on several cognitive domains like memory and executive function [72, 73], but no sex-specific analysis was conducted. Recurrent episodes of illness negatively affect cognition may through causing progressive damage to brain structure and function. It has been implied that multiple episodes could further dilate enlarged lateral ventricles [74] and reduce cerebellar vermis volume in patients with BD [75]. In addition, episode frequency was negatively related to left superior frontal cortex thickness in patient with BD [76]. Borgelt et al. also found that multi-episode bipolar subjects had lower regional activation across prefrontal-striatal-amygdala networks and lower glutamate and N-acetylaspartate concentrations in the anterior cingulate cortex related to those of first-episode bipolar subjects [77]. Yet, evidences have demonstrated that females appear to have a higher hazard of recurrence than males in BD [78, 79]. Based on the aforementioned, we speculated that recurrent episodes in female patients lead to secondary damage to brain parenchyma and secondary changes in cerebral functions, thereby causing the decline of verbal learning in BD. Herein, special attention should be paid to preventing or diminishing recurrence in female patients with BD. There are, however, no studies available on the mechanisms underlying sex differences in the effect of relapse frequency on cognition in BD or BD subtypes. Further systematic exploration in this aspect is necessary.

There were several limitations of the present study. First, this was a cross-sectional study, which cannot draw long-term effects of gender differences in cognitive functions. Further studies with larger samples and longitudinal designs are mandatory to address this concern. Second, we evaluated the sex-specific effect on cognition only in young adult patients with BD, so our results may not apply to other populations of patients with BD. Future studies employing different clinical populations are required to demonstrate whether sex-differential neurocognitive performance exists throughout the lifespan in patients with BD-II depression. Third, we did not simultaneously recruit patients with BD-I. Currently, the group differences in cognitive functions across BD subtypes have been shown; however, it is not yet known whether these group differences are mediated by gender. In the future, a systematic investigation of this aspect will be appropriate. Fourth, although we excluded subjects with a history of alcohol/substance abuse or dependence, we did not have data on cigarette or alcohol consumption, which could have been covariates in our analyses. Fifth, given the lack of demographic information on duration of illness and number of episodes that might affect cognition in HCs, we did not include these characteristics as covariates. Future studies should control for as many covariates as possible. Finally, the current study was a symptomatic study and was not involved in pathological mechanisms. ​The exact mechanism of the sex-specific impact on cognition of patients with BD-II depression requires further exploration.

## Conclusion

Our study demonstrated generalized cognitive deficits in unmedicated youths with BD-II depression. Moreover, male patients performed more serious cognitive impairment in attention/vigilance, verbal learning, and visual learning compared to female patients. Recurrent episodes of BD may aggravate the dysfunction of verbal learning in female patients. The present study provides an initial scientific rationale for sex-differential cognitive deficits in patients with BD-II depression. ​However, studies with larger samples, longitudinal designs and diverse clinical populations are necessary to increase effectiveness and universality of the results in the future.

### Electronic supplementary material

Below is the link to the electronic supplementary material.

Supplementary Material 1

## Data Availability

The datasets used during the current study are available from the corresponding author on reasonable request.

## Abbreviations

BD: Bipolar disorder
BD-I: BD subtype I
BD-II: BD subtype II
BDNF: Brain-derived neurotrophic factor
CRP: C-reactive protein
HCs: Healthy controls
MCCB: Measurement and Treatment Research to Improve Cognition in Schizophrenia (MATRICS) Consensus Cognitive Battery

## References

[1] WHO: World mental health report: transforming mental health for all. Geneva: World Health Organization, Geneva. (2022); 2022.

[2] Ilgen MA, Bohnert AS, Ignacio RV, McCarthy JF, Valenstein MM, Kim HM, Blow FC (2010). Psychiatric diagnoses and risk of suicide in veterans. Arch Gen Psychiatry.

[3] Nordentoft M, Mortensen PB, Pedersen CB (2011). Absolute risk of suicide after first hospital contact in mental disorder. Arch Gen Psychiatry.

[4] Gonda X, Pompili M, Serafini G, Montebovi F, Campi S, Dome P, Duleba T, Girardi P, Rihmer Z (2012). Suicidal behavior in bipolar disorder: epidemiology, characteristics and major risk factors. J Affect Disord.

[5] Plans L, Barrot C, Nieto E, Rios J, Schulze TG, Papiol S, Mitjans M, Vieta E, Benabarre A (2019). Association between completed suicide and bipolar disorder: a systematic review of the literature. J Affect Disord.

[6] Bourne C, Aydemir Ö, Balanzá-Martínez V, Bora E, Brissos S, Cavanagh JT, Clark L, Cubukcuoglu Z, Dias VV, Dittmann S (2013). Neuropsychological testing of cognitive impairment in euthymic bipolar disorder: an individual patient data meta-analysis. Acta Psychiatrica Scandinavica.

[7] Kurtz MM, Gerraty RT (2009). A meta-analytic investigation of neurocognitive deficits in bipolar illness: profile and effects of clinical state. Neuropsychology.

[8] Robinson LJ, Thompson JM, Gallagher P, Goswami U, Young AH, Ferrier IN, Moore PB (2006). A meta-analysis of cognitive deficits in euthymic patients with bipolar disorder. J Affect Disord.

[9] Bortolato B, Miskowiak KW, Köhler CA, Vieta E, Carvalho AF (2015). Cognitive dysfunction in bipolar disorder and schizophrenia: a systematic review of meta-analyses. Neuropsychiatr Dis Treat.

[10] Cullen B, Ward J, Graham NA, Deary IJ, Pell JP, Smith DJ, Evans JJ (2016). Prevalence and correlates of cognitive impairment in euthymic adults with bipolar disorder: a systematic review. J Affect Disord.

[11] Goodwin GM, Martinez-Aran A, Glahn DC, Vieta E (2008). Cognitive impairment in bipolar disorder: neurodevelopment or neurodegeneration? An ECNP expert meeting report. Eur Neuropsychopharmacology: J Eur Coll Neuropsychopharmacol.

[12] Quraishi S, Frangou S (2002). Neuropsychology of bipolar disorder: a review. J Affect Disord.

[13] Gillissie ES, Lui LMW, Ceban F, Miskowiak K, Gok S, Cao B, Teopiz KM, Ho R, Lee Y, Rosenblat JD (2022). Deficits of social cognition in bipolar disorder: systematic review and meta-analysis. Bipolar Disord.

[14] Chaves OC, Lombardo LE, Bearden CE, Woolsey MD, Martinez DM, Barrett JA, Miller AL, Velligan DI, Glahn DC (2011). Association of clinical symptoms and neurocognitive performance in bipolar disorder: a longitudinal study. Bipolar Disord.

[15] Santos JL, Aparicio A, Bagney A, Sánchez-Morla EM, Rodríguez-Jiménez R, Mateo J, Jiménez-Arriero M (2014). A five-year follow-up study of neurocognitive functioning in bipolar disorder. Bipolar Disord.

[16] Vlad M, Raucher-Chéné D, Henry A, Kaladjian A (2018). Functional outcome and social cognition in bipolar disorder: is there a connection?. Eur Psychiatry: J Association Eur Psychiatrists.

[17] Martinez-Aran A, Scott J, Colom F, Torrent C, Tabares-Seisdedos R, Daban C, Leboyer M, Henry C, Goodwin GM, Gonzalez-Pinto A (2009). Treatment nonadherence and neurocognitive impairment in bipolar disorder. J Clin Psychiatry.

[18] Baune BT, Malhi GS (2015). A review on the impact of cognitive dysfunction on social, occupational, and general functional outcomes in bipolar disorder. Bipolar Disord.

[19] Bearden CE, Shih VH, Green MF, Gitlin M, Sokolski KN, Levander E, Marusak S, Hammen C, Sugar CA, Altshuler LL (2011). The impact of neurocognitive impairment on occupational recovery of clinically stable patients with bipolar disorder: a prospective study. Bipolar Disord.

[20] Carrus D, Christodoulou T, Hadjulis M, Haldane M, Galea A, Koukopoulos A, Kumari V, Frangou S (2010). Gender differences in immediate memory in bipolar disorder. Psychol Med.

[21] Bücker J, Popuri S, Muralidharan K, Kozicky JM, Baitz HA, Honer WG, Torres IJ, Yatham LN (2014). Sex differences in cognitive functioning in patients with bipolar disorder who recently recovered from a first episode of mania: data from the Systematic Treatment Optimization Program for early mania (STOP-EM). J Affect Disord.

[22] Barrett SL, Kelly C, Bell R, King DJ (2008). Gender influences the detection of spatial working memory deficits in bipolar disorder. Bipolar Disord.

[23] Xu X, Xiang H, Qiu Y, Teng Z, Li S, Huang J, Chen J, Tang H, Jin K, Jiang L (2021). Sex differences in cognitive function of first-diagnosed and drug-naïve patients with bipolar disorder. J Affect Disord.

[24] Bora E, Yücel M, Pantelis C, Berk M (2011). Meta-analytic review of neurocognition in bipolar II disorder. Acta Psychiatrica Scandinavica.

[25] Cotrena C, Damiani Branco L, Ponsoni A, Samamé C, Milman Shansis F, Paz Fonseca R (2020). Executive functions and memory in bipolar disorders I and II: new insights from meta-analytic results. Acta Psychiatrica Scandinavica.

[26] Bora E (2018). Neurocognitive features in clinical subgroups of bipolar disorder: a meta-analysis. J Affect Disord.

[27] Dickinson T, Becerra R, Coombes J (2017). Executive functioning deficits among adults with bipolar disorder (types I and II): a systematic review and meta-analysis. J Affect Disord.

[28] Martínez-Arán A, Vieta E, Reinares M, Colom F, Torrent C, Sánchez-Moreno J, Benabarre A, Goikolea JM, Comes M, Salamero M (2004). Cognitive function across manic or hypomanic, depressed, and euthymic states in bipolar disorder. Am J Psychiatry.

[29] Malhi GS, Ivanovski B, Hadzi-Pavlovic D, Mitchell PB, Vieta E, Sachdev P (2007). Neuropsychological deficits and functional impairment in bipolar depression, hypomania and euthymia. Bipolar Disord.

[30] Hermens DF, Jamieson D, Fitzpatrick L, Sacks DD, Iorfino F, Crouse JJ, Guastella AJ, Scott EM, Hickie IB, Lagopoulos J. Sex differences in fronto-limbic white matter tracts in youth with mood disorders. Psychiatry and Clinical Neurosciences. 2022. 10.1111/pcn.13440 35730893

[31] Young RC, Biggs JT, Ziegler VE, Meyer DA (1978). A rating scale for mania: reliability, validity and sensitivity. Br J Psychiatry: J Mental Sci.

[32] Bo Q, Mao Z, Li X, Wang Z, Wang C, Ma X (2017). Use of the MATRICS consensus cognitive battery (MCCB) to evaluate cognitive deficits in bipolar disorder: a systematic review and meta-analysis. PLoS ONE.

[33] Yatham LN, Torres IJ, Malhi GS, Frangou S, Glahn DC, Bearden CE, Burdick KE, Martínez-Arán A, Dittmann S, Goldberg JF (2010). The International Society for Bipolar Disorders-Battery for Assessment of Neurocognition (ISBD-BANC). Bipolar Disord.

[34] Shi C, Kang L, Yao S, Ma Y, Li T, Liang Y, Cheng Z, Xu Y, Shi J, Xu X (2015). The MATRICS Consensus Cognitive Battery (MCCB): co-norming and standardization in China. Schizophr Res.

[35] Zhu Y, Womer FY, Leng H, Chang M, Yin Z, Wei Y, Zhou Q, Fu S, Deng X, Lv J (2019). The Relationship between Cognitive Dysfunction and Symptom dimensions Across Schizophrenia, bipolar disorder, and major depressive disorder. Front Psychiatry.

[36] Teng Z, Wang L, Li S, Tan Y, Qiu Y, Wu C, Jin K, Chen J, Huang J, Tang H (2021). Low BDNF levels in serum are associated with cognitive impairments in medication-naïve patients with current depressive episode in BD II and MDD. J Affect Disord.

[37] Chen P, Chen G, Zhong S, Chen F, Ye T, Gong J, Tang G, Pan Y, Luo Z, Qi Z (2022). Thyroid hormones disturbances, cognitive deficits and abnormal dynamic functional connectivity variability of the amygdala in unmedicated bipolar disorder. J Psychiatr Res.

[38] Zhong S, Lai S, Yue J, Wang Y, Shan Y, Liao X, Chen J, Li Z, Chen G, Chen F (2020). The characteristic of cognitive impairments in patients with bipolar II depression and its association with N-acetyl aspartate of the prefrontal white matter. Annals Translational Med.

[39] Chang YH, Wang TY, Lee SY, Chen SL, Huang CC, Chen PS, Yang YK, Hong JS, Lu RB (2018). Memory impairment and plasma BDNF correlates of the BDNF Val66Met polymorphism in patients with bipolar II disorder. Front Genet.

[40] Young AH (2014). The effects of HPA axis function on cognition and its implications for the pathophysiology of bipolar disorder. Harv Rev Psychiatry.

[41] Lai S, Zhong S, Zhang Y, Wang Y, Zhao H, Chen G, Chen F, Shen S, Huang H, Jia Y (2021). Association of altered thyroid hormones and neurometabolism to cognitive dysfunction in unmedicated bipolar II depression. Prog Neuro-psychopharmacol Biol Psychiatry.

[42] Huang MH, Chan YE, Chen MH, Hsu JW, Huang KL, Li CT, Tsai SJ, Bai YM, Su TP. Pro-inflammatory cytokines and cognitive dysfunction among patients with bipolar disorder and major depression. Psychiatry and clinical neurosciences; 2022. 10.1111/pcn.13433 35674415

[43] Zazula R, Dodd S, Dean OM, Berk M, Bortolasci CC, Verri WA, Vargas HO, Nunes SOV (2022). Cognition-immune interactions between executive function and working memory, tumour necrosis factor-alpha (TNF-alpha) and soluble TNF receptors (sTNFR1 and sTNFR2) in bipolar disorder. World J Biol Psychiatry: Official J World Federation Soc Biol Psychiatry.

[44] Hua MH, Chen MH, Hsu JW, Huang KL, Tsai SJ, Li CT, Bai YM (2021). Proinflammatory cytokine dysregulation and cognitive dysfunction among patients with remitted bipolar I and II disorders. J Affect Disord.

[45] Lu RB, Wang TY, Lee SY, Chang YH, Chen SL, Tsai TY, Chen PS, Huang SY, Tzeng NS, Lee IH (2021). Add-on memantine may improve cognitive functions and attenuate inflammation in middle- to old-aged bipolar II disorder patients. J Affect Disord.

[46] Sanchez-Autet M, Arranz B, Safont G, Sierra P, Garcia-Blanco A, de la Fuente L, Garriga M, García-Portilla MP (2018). Gender differences in C-reactive protein and homocysteine modulation of cognitive performance and real-world functioning in bipolar disorder. J Affect Disord.

[47] Solé B, Varo C, Torrent C, Montejo L, Jiménez E, Bonnin CDM, Clougher D, Verdolini N, Amoretti S, Piazza F (2022). Sex differences in neurocognitive and psychosocial functioning in bipolar disorder. J Affect Disord.

[48] Jogia J, Dima D, Frangou S (2012). Sex differences in bipolar disorder: a review of neuroimaging findings and new evidence. Bipolar Disord.

[49] Zimmerman ME, DelBello MP, Getz GE, Shear PK, Strakowski SM (2006). Anterior cingulate subregion volumes and executive function in bipolar disorder. Bipolar Disord.

[50] Yoshimura Y, Okamoto Y, Onoda K, Okada G, Toki S, Yoshino A, Yamashita H, Yamawaki S (2014). Psychosocial functioning is correlated with activation in the anterior cingulate cortex and left lateral prefrontal cortex during a verbal fluency task in euthymic bipolar disorder: a preliminary fMRI study. J Neuropsychiatry Clin Neurosci.

[51] Bertocci MA, Bebko GM, Mullin BC, Langenecker SA, Ladouceur CD, Almeida JR, Phillips ML (2012). Abnormal anterior cingulate cortical activity during emotional n-back task performance distinguishes bipolar from unipolar depressed females. Psychol Med.

[52] Farrow TF, Whitford TJ, Williams LM, Gomes L, Harris AW (2005). Diagnosis-related regional gray matter loss over two years in first episode schizophrenia and bipolar disorder. Biol Psychiatry.

[53] Sassi RB, Brambilla P, Hatch JP, Nicoletti MA, Mallinger AG, Frank E, Kupfer DJ, Keshavan MS, Soares JC (2004). Reduced left anterior cingulate volumes in untreated bipolar patients. Biol Psychiatry.

[54] Kaur S, Sassi RB, Axelson D, Nicoletti M, Brambilla P, Monkul ES, Hatch JP, Keshavan MS, Ryan N, Birmaher B (2005). Cingulate cortex anatomical abnormalities in children and adolescents with bipolar disorder. Am J Psychiatry.

[55] Delvecchio G, Ciappolino V, Perlini C, Barillari M, Ruggeri M, Altamura AC, Bellani M, Brambilla P (2019). Cingulate abnormalities in bipolar disorder relate to gender and outcome: a region-based morphometry study [corrected]. Eur Arch Psychiatry Clin NeuroSci.

[56] Fornito A, Yücel M, Wood SJ, Bechdolf A, Carter S, Adamson C, Velakoulis D, Saling MM, McGorry PD, Pantelis C (2009). Anterior cingulate cortex abnormalities associated with a first psychotic episode in bipolar disorder. Br J Psychiatry: J Mental Sci.

[57] Mitchell RH, Metcalfe AW, Islam AH, Toma S, Patel R, Fiksenbaum L, Korczak D, MacIntosh BJ, Goldstein BI. Sex differences in brain structure among adolescents with bipolar disorder. *Bipolar disorders* 2018. 10.1111/bdi.12663 29956452

[58] Lai MC, Lombardo MV, Suckling J, Ruigrok AN, Chakrabarti B, Ecker C, Deoni SC, Craig MC, Murphy DG, Bullmore ET (2013). Biological sex affects the neurobiology of autism. Brain.

[59] Frey BN, Dias RS (2014). Sex hormones and biomarkers of neuroprotection and neurodegeneration: implications for female reproductive events in bipolar disorder. Bipolar Disord.

[60] Hwang WJ, Lee TY, Kim NS, Kwon JS. The role of Estrogen Receptors and their signaling across Psychiatric disorders. Int J Mol Sci 2020, 22(1). 10.3390/ijms22010373 PMC7794990 33396472

[61] Sellers K, Raval P, Srivastava DP (2015). Molecular signature of rapid estrogen regulation of synaptic connectivity and cognition. Front Neuroendocr.

[62] Hara Y, Waters EM, McEwen BS, Morrison JH (2015). Estrogen effects on cognitive and synaptic Health over the Lifecourse. Physiol Rev.

[63] Liang J, Shang Y (2013). Estrogen and cancer. Annu Rev Physiol.

[64] Marchant I, Stojanova J, Acevedo L, Olivero P (2022). Estrogen rapid effects: a window of opportunity for the aging brain?. Neural Regeneration Res.

[65] Sherwin BB (2002). Estrogen and cognitive aging in women. Trends Pharmacol Sci.

[66] Ingraham HA, Herber CB, Krause WC (2022). Running the Female Power Grid Across Lifespan through Brain Estrogen Signaling. Annu Rev Physiol.

[67] Kim K, Joo YY, Ahn G, Wang HH, Moon SY, Kim H, Ahn WY, Cha J (2022). The sexual brain, genes, and cognition: a machine-predicted brain sex score explains individual differences in cognitive intelligence and genetic influence in young children. Hum Brain Mapp.

[68] Laing KR, Mitchell D, Wersching H, Czira ME, Berger K, Baune BT (2012). Brain-derived neurotrophic factor (BDNF) gene: a gender-specific role in cognitive function during normal cognitive aging of the MEMO-Study?. Age (Dordrecht Netherlands).

[69] Zhang ZZ, Chen J, Luo BL, Ni MZ, Liu X, Zeng LP, Yang QG, Wang F, Chen GH (2022). Maternal inflammation induces spatial learning and memory impairment in the F1 and F2 generations of mice via sex-specific epigenetic mechanisms. Brain Res Bull.

[70] Keshavarzi A, Eftekharian MM, Komaki A, Omrani MD, Kholghi Oskooei V, Taheri M, Ghafouri-Fard S (2019). Sexual dimorphism in up-regulation of suppressors of cytokine signaling genes in patients with bipolar disorder. BMC Psychiatry.

[71] Slaney C, Sallis HM, Jones HJ, Dardani C, Tilling K, Munafò MR, Davey Smith G, Mahedy L, Khandaker GM (2023). Association between inflammation and cognition: Triangulation of evidence using a population-based cohort and mendelian randomization analyses. Brain Behav Immun.

[72] Elshahawi HH, Essawi H, Rabie MA, Mansour M, Beshry ZA, Mansour AN (2011). Cognitive functions among euthymic bipolar I patients after a single manic episode versus recurrent episodes. J Affect Disord.

[73] Nehra R, Chakrabarti S, Pradhan BK, Khehra N (2006). Comparison of cognitive functions between first- and multi-episode bipolar affective disorders. J Affect Disord.

[74] Strakowski SM, DelBello MP, Zimmerman ME, Getz GE, Mills NP, Ret J, Shear P, Adler CM (2002). Ventricular and periventricular structural volumes in first- versus multiple-episode bipolar disorder. Am J Psychiatry.

[75] Mills NP, Delbello MP, Adler CM, Strakowski SM (2005). MRI analysis of cerebellar vermal abnormalities in bipolar disorder. Am J Psychiatry.

[76] Achalia R, Raju VB, Jacob A, Nahar A, Achalia G, Nagendra B, Kaginalkar V, Choudhary S, Venkatasubramanian G, Rao NP (2020). Comparison of first-episode and multiple-episode bipolar disorder: a surface-based morphometry study. Psychiatry Res Neuroimaging.

[77] Borgelt L, Strakowski SM, DelBello MP, Weber W, Eliassen JC, Komoroski RA, Chu WJ, Welge JA, Blom TJ, Rummelhoff E (2019). Neurophysiological effects of multiple mood episodes in bipolar disorder. Bipolar Disord.

[78] Suominen K, Mantere O, Valtonen H, Arvilommi P, Leppämäki S, Isometsä E (2009). Gender differences in bipolar disorder type I and II. Acta Psychiatrica Scandinavica.

[79] Diflorio A, Jones I (2010). Is sex important? Gender differences in bipolar disorder. Int Rev Psychiatry (Abingdon).

