# Supplementary material for: Sex-differential cognitive performance on MCCB of youth with BD-II depression
Source: BMC Psychiatry. 2024 May 7;24:345. doi: 10.1186/s12888-024-05701-7 (PMC11077867; doi:10.1186/s12888-024-05701-7)
Supplement: Supplementary file 1 — Supplementary Material 1 [file 12888_2024_5701_MOESM1_ESM.docx]

**Supplementary material**

**Sex-differential cognitive performance on MCCB of youth with BD-II depression**

**Contents:**

Figure S1. Pearson correlation coefficients between attention/vigilance, verbal learning, visual learning and composite in patients with BD-II depression.

Figure S2. Pearson correlation coefficients between attention/vigilance, verbal learning, visual learning and composite in healthy controls.


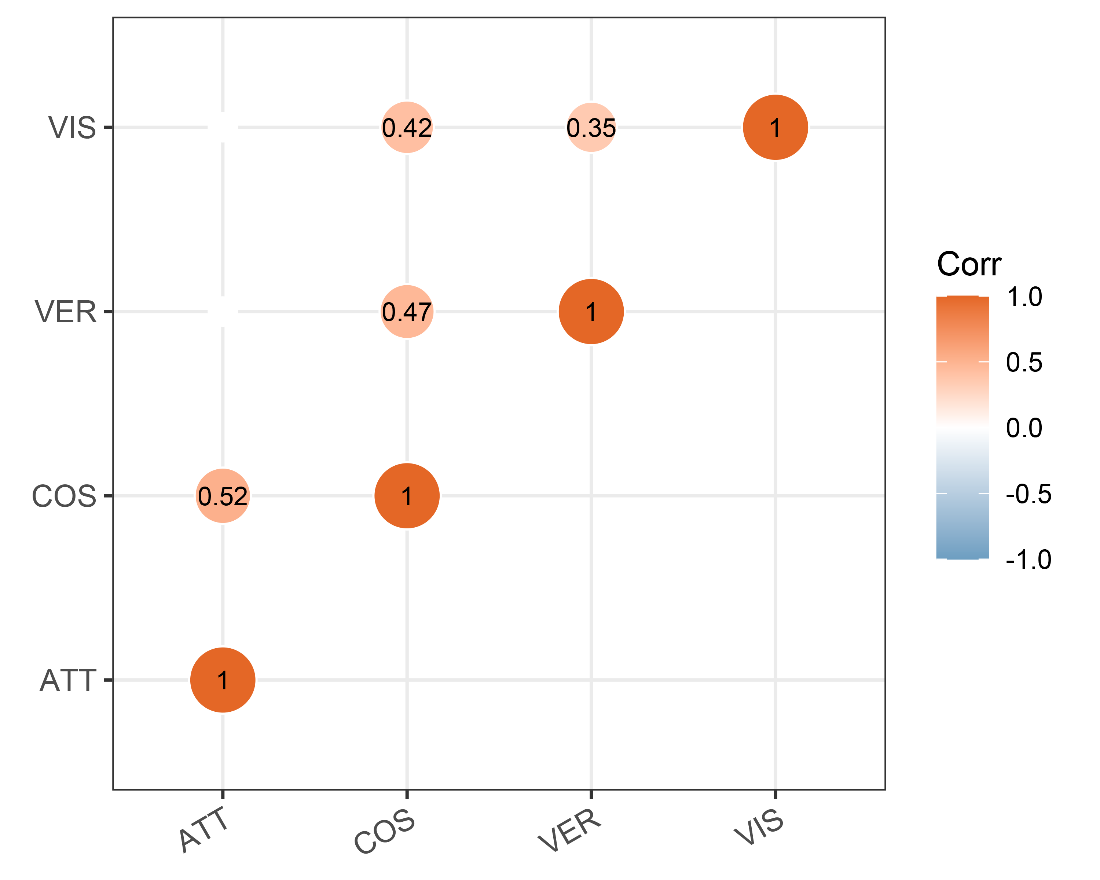


Figure S1. Pearson correlation coefficients between attention/vigilance, verbal learning, visual learning and composite in patients with BD-II depression. ATT = attention/vigilance; VER = verbal learning; VIS = visual learning; COS = composite. *P*-values were Bonferroni corrected.


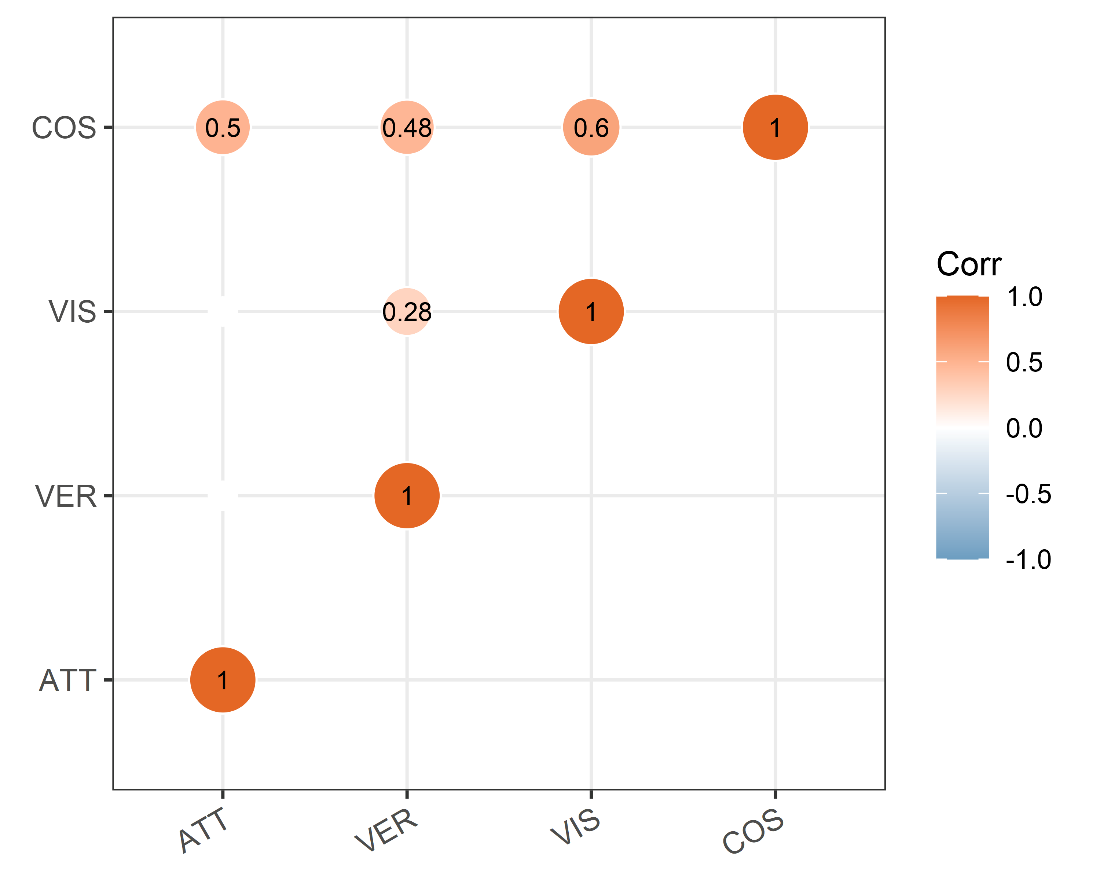


Figure S2. Pearson correlation coefficients between attention/vigilance, verbal learning, visual learning and composite in healthy controls. ATT = attention/vigilance; VER = verbal learning; VIS = visual learning; COS = composite. *P*-values were Bonferroni corrected.
